# Accelerometer-Based Wheel Odometer for Kinematics Determination

**DOI:** 10.3390/s21041327

**Published:** 2021-02-13

**Authors:** Ahmed A. Youssef, Naif Al-Subaie, Naser El-Sheimy, Mohamed Elhabiby

**Affiliations:** 1 Department of Geomatics Engineering, Schulich School of Engineering, University of Calgary, Calgary, AB T2N 1N4, Canada; elsheimy@ucalgary.ca; 2 Saudi Ministry of Education, Riyadh 14811, Saudi Arabia; alsubanaif@gmail.com; 3 Public Works Department, Faculty of Engineering, Ain Shams University, Cairo 11566, Egypt; mmelhabiby@eng.asu.edu.eg

**Keywords:** wheel odometer, wheel encoders, peak-valley relationship, micro-electro-mechanical systems, accelerometers, accelerometer-based wheel odometry

## Abstract

Various high budget industries that utilize wheel-based vehicles rely on wheel odometry as an integral aspect of their navigation process. This research introduces a low-cost alternative for typical wheel encoders that are typically used to determine the on-track speed of vehicles. The proposed system is referred to as an Accelerometer-based Wheel Odometer for Kinematics determination (AWOK). The AWOK system comprises just a single axis accelerometer mounted radially at the center of any given wheel. The AWOK system can provide direct distances instead of just velocities, which are provided by typical wheel speedometers. Hence, the AWOK system is advantageous in comparison to typical wheel odometers. Besides, the AWOK system comprises a simple assembly with a highly efficient data processing algorithm. Additionally, the AWOK system provides a high capacity to handle high dynamics in comparison to similar approaches found in previous related work. Furthermore, the AWOK system is not affected by the inherited stochastic errors in micro-machined electro-mechanical systems (MEMS) inertial sensors, whether short-term or long-term errors. Above all, the AWOK system reported a relative accuracy of 0.15% in determining the distance covered by a car.

## 1. Introduction

Wheel odometry is considered a huge aspect for various industries that are dependent on wheel-based vehicles. Such applications include mobile mapping, self-driving cars, automotive industry safety applications, and various other high budget industries [1,2,3]. Consequently, it can be postulated that there is a considerable major research aspect to provide a more precise and cheaper alternative for the typical wheel encoders that are currently used in those industries. Hence, this research introduces a low-cost alternative system for typical wheel encoders, which provides a comparable level of accuracy. Besides, the proposed system is advantageous in its flexibility for integration into different navigation sensor fusion architectures by providing easily accessible and interoperable data.

To be specific, the system proposes an effective solution for state-of-the-art research aspects in the automotive industry, especially for the current research in self-driving cars. Autonomous features are expected to increase within the automotive industry, and the revenue from features is expected to reach $250 billion by 2030 [4]. One of the major research aspects in autonomous self-driving cars is navigation.

It is highly plausible that the proposed system can shift the self-driving car industry to an entirely different paradigm, especially for the navigation and environment perception aspects, at extremely high precision and low cost in comparison to the currently proposed solutions. The contributions of the system can speed up the research in the self-driving cars industry to reach level 5, which is fully autonomous self-driven vehicles, in a shorter span of time than expected in the current literature [5].

Typically, cars navigate the urban environment, which hinders the performance of Global Navigation Satellite Systems (GNSS) due to multipath errors and signal blockage within urban canyons. Consequently, the GNSS is integrated with inertial measurement units (IMUs) to remedy the problem of GNSS signal degradation and/or blockage [2,6]. However, low-cost automotive-grade IMUs endure a multitude of long-term stochastic errors. Consequently, in the case of long operation periods and degraded GNSS solution, the overall navigation solution would deteriorate. Consequently, car navigation systems are augmented through the integration of wheel odometry measurements and implementing some motion constraints for wheel-based vehicles (such as non-holonomic constraints (NHC) and zero velocity updates (ZUPT)). Hence, it is a crucial aspect of integrating the wheel odometry, is to ensure accurate velocity and distance measurements.

The introduced concept of using accelerometers as wheel odometers in wheel-based vehicles is based on an analogy to some pedestrian dead reckoning (PDR) applications. In some PDR applications, accelerometer signals are acquired and analyzed for the patterns generated by pedestrian motion. Afterwards, a series of context analysis is performed to deduce the activity held by the pedestrian user. Nonetheless, PDR is a vast research area with its own problems and objectives. However, one can make use of the analogy of identifying motion kinematics via the analysis of the repetitive pattern within a platform motion.

Nevertheless, the concept referred to hereafter as Accelerometer-based Wheel Odometer for Kinematics Determination (AWOK) utilizes an assembly of extremely low-cost consumer-grade accelerometers that are mounted to the wheels of a wheel-based vehicle. The mounted accelerometers are used to detect the covered distance by the wheel-based vehicle.

This adoption of AWOK provides distances instead of velocities, which mathematically is significantly effective in terms of the induced error budget within any navigation solution acquired. To clarify, velocities are integrated over time to acquire the travelled distance. Under the assumption of erroneous velocity measurements, the resulting integrated distance would endure an error whose magnitude is a linear function with time.

Moreover, it is noted that the traditional wheel encoders have a low bandwidth in comparison to inertial sensors, namely, accelerometers. Hence, the bandwidth of the AWOK algorithm would be larger than that of any conventional wheel-based encoders, which is realized at a relatively low cost, whereas AWOK deploys an assembly of micro-electro-mechanical systems (MEMS) accelerometers.

Additionally, AWOK is immune to the inherited stochastic errors, which are characteristic of low-cost MEMS-based inertial measurement sensors. To clarify, as per the AWOK system design and assembly, it can be discerned that the AWOK incorporates an assembly of low-cost MEMS-based inertial sensors. Nonetheless, the inertial sensors are mounted such that they are subjected to an extremely high dynamics level that surpasses the magnitude of all the stochastic errors, which might be found in such grade of inertial sensors. Moreover, the data processing algorithm is designed to overcome potential effects induced by such stochastic errors within the AWOK sensors assembly, which is described in detail hereafter.

In Section 2, related work upon state-of-the-art techniques in accelerometer-based odometry, along with the analogous step detection methods utilized in PDR navigation. In Section 3, the mathematical background governing the operation of the AWOK system is covered by explaining the physical phenomenon for the acquired AWOK raw data. In Section 4, the AWOK system setup is discussed, including the design parameters that should be considered while designing and using the AWOK system. Besides, Section 4 covers the system design and AWOK algorithm’s detailed description. Section 5 discusses the experimental validation of the AWOK system, whereas Section 5 covers the detailed experiment description, results, and experimental observations. Section 6 presents the discussion and the conclusions on whether the AWOK algorithm meets its premise. Finally, Section 7 shows some recommendations for future work that should be addressed to further enhance the performance of the AWOK system.

## 2. Related Work

### 2.1. State-of-the-Art Techniques in Accelerometer-Based Odometry

Croyle et al. (2001) proposed an improved vehicle navigation system using multiple axes accelerometers [7]. The proposed navigation system did not utilize any wheel-mounted accelerometers; however, it was an attempt to utilize an accelerometer unconventionally to acquire a full navigation solution. The improved system utilizes multiple accelerometers, which are mounted along orthogonal axes. To specify, a set of three accelerometers are mounted on any given car to constitute the improved navigation system. A longitudinal accelerometer is mounted along with the nose to the rear bumper direction of the car. Whereas a lateral accelerometer is mounted such that its sensitive axis is along the left to right side direction of the car. Besides, an optional accelerometer is mounted along the vertical direction and orthogonal to the sensitive axis of the longitudinal and lateral accelerometers. The longitudinal accelerometer is used to determine the incremental change in velocity, which can be integrated to get the on-track displacement of the vehicle. While the lateral accelerometer measures the centrifugal acceleration acting upon the car, from which the rate of turn can be determined. Consequently, the heading of the vehicle can be computed. Whereas the vertical accelerometer is used to measure the pitch angle influencing the vehicle.

Pierbon filed a U.S. patent (2006), in which a device for detecting the position of a vehicle wheel was proposed [8]. The proposed device is used to determine the position of a wheel on a vehicle and is designed particularly to be used in a tire pressure monitoring system for motor wheel-based vehicles. Typical tire pressure monitoring systems incorporate a set of pressure sensors mounted within each tire. Whereby each tire sensor is fitted with a transmitter to send pressure measurements to the vehicle’s control unit. Hence, if there would be a persistent problem, the vehicle driver would be informed promptly. However, the main issue is identifying which wheel has the problem depending on the received signal from the pressure sensors. Hence, the main purpose of the invention was to provide a device that can identify whether the signal from the malfunctioning tire is coming from the left or the right wheel in a self-contained manner. Whereby, the device can determine which tire has the problem without the need for exchanging information with the on-board control unit of the car. The device comprises two accelerometers, which are placed within an electric board, along with a tire pressure sensor. The accelerometers are placed such that their sensitive axes are pointed with a known shift angle between them. Whereas the offset angle between the accelerometers’ sensitive axes is neither 0° nor 180°, to avoid confusion of the phase angle with the start or end of the motion. Moreover, the accelerometers are mounted to the tire such that their sensitive axis lies within the plane of the wheel, and the accelerometers are subject to accelerations that occur within the vertical plane. As the wheel rotates, each accelerometer would register a component of the acceleration due to gravity. By comparing both accelerometers’ readouts, there should exist a phase shift between the acquired acceleration signals due to the physical angle at which the accelerometers are mounted. The investor assumes that the car’s motion direction is known and is transmitted to the board connected to the accelerometers. Consequently, by examining the sign of the phase shift between the two accelerometers and knowing the vehicle’s direction of motion, the on-board processing algorithm can detect whether the wheel to which the device is mounted lies on the right or the left of the vehicle. Therefore, if the tire pressure resides to a value below a predefined threshold, the wheel can then send such information to the main vehicle control unit, along with information indicating which wheel does the problem exists. However, the proposed device was not attempted for any use for a navigation application and did not consider the implications that typically occur in such context.

Huang and Wang (2011) introduced a wheel-mounted accelerometers assembly to be mounted for navigation applications [9]. The wheel-mounted accelerometers are chosen to be low-cost MEMS accelerometers. The premise of such publication was utilizing the wheel-mounted accelerometers to overcome the poor accuracy of the MEMS accelerometers. The wheel-mounted accelerometers are used to detect the angular velocity and rotation of the wheel to which the accelerometer is mounted, with a high degree of precision. The angular velocity and wheel rotation angles could be determined by computing the arctan of the ratio between the accelerometer, which is mounted along the radial direction of the wheel, and the accelerometer, which is mounted along the tangential direction of the wheel. Afterwards, the distance cover by the wheel could be determined. Nevertheless, the assembly was tested using a bicycle over a trajectory of length 967 m. Besides, the distances acquired from the wheel-mounted accelerometers were integrated with the measurements acquired from a magnetometer into a navigation filter. Hence, a final two-dimensional (2D) navigation solution was acquired. The integrated results of the wheel-mounted accelerometers and the magnetometer reached an error of 15 m over the trajectory length. However, the results are inconclusive as they did not differentiate between the errors were induced by using the wheel-mounted accelerometers and the errors caused by the magnetometer. Besides, the assembly was only tested for navigation of only bicycles, which typically is subjected to extremely low-dynamics in comparison to other wheel-based vehicles, such as cars and unmanned ground vehicles (UGVs).

Coulter et al. (2012) published an article that introduced a physical activity monitoring system for use on a wheelchair [10]. The monitoring system was intended for patients with injuries related to their spinal cord who use wheelchairs for mobility. Patients with spinal cord injuries are more susceptible to physical inactivity; hence, the importance of their research lies in determining the level of physical activity of the patients in relation to their injuries and their overall well-being. Nevertheless, the physical activity monitoring system comprised a triaxial accelerometer placed on the wheel of a wheelchair, along with a data processing algorithm. Like the approach introduced by Huang and Wang (2011), the two accelerometers, which lie within the plane of the wheel, are used to compute the angle of rotation of the wheel [9,10]. Afterwards, the number of revolutions, distance, speed, and duration of motion of the wheelchair can be derived. The system was tested against a reference acquired by videotaping the length along which the test was held. The reference wheel revolutions and covered distance were computed using colour-coded targets, which were mounted on the wheelchair wheel. Consequently, the revolutions are determined by tracking those colour-coded targets. It is noted that the testing process was performed for indoor and outdoor usage of the wheelchair. Besides, the testing included portions where the wheelchairs were moved forwards and backwards, and the used wheelchairs varied between manually propelled and automatically propelled wheelchairs. The study reported that the wheel revolutions could be acquired with a mean difference of 0.002 ± 0.016 revolutions with respect to the reference videotaped wheel revolutions. Besides, the error within the absolute wheel rotation angle was 0.006 ± 3.853°. At the same time, the duration of wheelchair movement was acquired with a mean difference of 0.647 s. Finally, it is noted that the results were validated using intra-class correlation (ICC) analysis and a Bland–Altman plot, with confidence intervals of 95% for each of the wheel revolutions, absolute angle, and duration of the movement.

A similar system was also introduced by Hiremath et al. (2013); however, the system utilizes a gyroscope instead of accelerometers to estimate the speeds and distances travelled by wheelchair users [11]. The system is referred to as a wireless gyroscope-based wheel rotation monitor (G-WRM). The G-WRM system can be used to capture dynamics encountered with regular wheel propulsion and wheelchair sports. The G-WRM provides its user with real-time feedback through a smartphone. The speeds and the distances were validated using ICC and Bland–Altman plots against the reference data. The testing process involved testing the G-WRM against a Computer Numerical Control (CNC) lathe to determine its precision in deriving the number of wheel revolutions. Another test was performed using the G-WRM against a double drum, which was used to check the performance of the G-WRM at different speeds and for simulating uneven terrain, which is typically encountered while using wheelchairs. A distance test was performed for the G-WRM against distances measured using measurement tape for 54 trials. Furthermore, the G-WRM was tested for hand cycling, battery life, and wireless data transmission. It was concluded that the G-WRM percentage errors for angular velocities, linear speeds, and distances were less than 3% for all the test trials. Besides, the G-WRM reported a battery life of 35 h in wireless mode and 139 h in secure digital card mode, and a maximum data loss of 0.3%. Despite having extremely promising results, the G-WRM was only intended for wheelchairs and their expected dynamics.

Sonenblum et al. (2012) introduced a similar accelerometer-based method to measure the use of manual wheelchairs [12]. In contrast, a biaxial accelerometer was mounted to the wheel of a manually operated wheelchair. In their approach, they computed the rotation angle of the wheel by computing the arctan of the ratio between the two sensitive axes of the accelerometer. Afterwards, the rotation angle was then differentiated to compute the velocity of the wheelchair. Nonetheless, the main objective of their research was to identify whether the wheelchair was in motion; hence, the acquired velocity was compared against a predefined threshold to define the state of the wheelchair. Besides, the invention was limited to an application with extremely low and predetermined dynamics, which limits the implementation of their system to higher and more versatile navigation applications.

Gersdorf and Frese (2013) have also demonstrated a Kalman filter algorithm for using a wheel mounted inertial sensor [13]. The paper discusses an extended Kalman filter (EKF), which is designed to incorporate the measurements from a wheel-mounted inertial measurement unit (IMU). The IMU includes two accelerometers and a single-axis gyroscope as an alternative for classical odometry sensing. The bi-axial accelerometers are mounted within the plane of the vehicle’s wheel, and the gyroscope’s sensitive axis is set to be orthogonal to the plane of the wheel. Hence, the gyroscope would detect the angular rate of the wheel. In comparison, the accelerometers detect the gravity components along each axis, besides the centrifugal acceleration and tangential acceleration components of the wheel. The algorithm implements an EKF, in which the biaxial accelerations are used as a process model, and the measurements from the gyroscope, along with the accelerometer data, are used within the measurement model. In the EKF, the process noise within the accelerometer measurements is assumed to be a random walk. At the same time, the measurement noise in the gyroscope is assumed to be white noise, with zero mean and Gaussian distribution. The EKF algorithm showed promising results, in which a series of experiments were held. The experiments tested the algorithm for uneven terrain, high speed, wheel rotation angle truth in comparison to reference odometer, and long distances. The EKF algorithm was capable of handling uneven terrain, which caused acceleration spikes within the range of −40 to 15 m/$s^{2}$. Furthermore, the EKF algorithm reported handling speeds up to 4 m/s, despite having an accelerometer and the gyroscope was saturated. Moreover, the algorithm was tested for a long distance of 4 km, in which the acquired number of wheel revolutions from the integrated EKF solution reached 0.4 revolutions in error with respect to the reference number of revolutions covered by the vehicle (i.e., bicycle). Nevertheless, the system has some limitations, including the use of multiple sensors to acquire the odometry data (i.e., distances) and limited dynamics where the experimental validation of the EKF fusion was done using a bicycle and a walker as the testing vehicles.

To sum up, it has been shown that, in relevant and close implementation of wheel-mounted inertial sensors as odometers, the systems are designed and tested for extremely low-dynamics applications. Besides, some of the results are inconclusive in terms of the expected accuracy of the system.

### 2.2. Pedestrian Dead Reckoning

As mentioned earlier, the AWOK system relies, in concept, on the ideas typically encountered within PDR algorithms. To be specific, the AWOK system emulates the step detection portion within PDR algorithms. As stated by Lee et al. (2015), conventional methods of step detection are classified into thresholds, peak detection, auto-correlation, or spectral analysis of the acquired acceleration signal from whichever human-carriable inertial sensors [14].

Nonetheless, peak detection-based methods are the most relevant to the implementation of the AWOK system to acquire the dynamics of wheel-based sensors. To clarify, peak detection-based methods make use of the periodic behaviour of human stepping motion [15,16,17,18,19,20,21]. The peak detection algorithms require identifying local peaks and valleys within any given acceleration signal and counting the number of said peaks or valleys. Accordingly, the number of steps can be derived thereafter—however, such algorithms are required to be proofed against over-counting of the steps. Consequently, different measures are taken to avoid over-counting, including comparing each acquired peak or valley against a magnitude threshold [15,16,19,21]. Besides, the time between any two consecutive peaks can be compared against a temporal threshold [15,16,19,21], depending on knowing which kind of activity is carried out by the pedestrian user, such as walking or running. Another measure to prevent steps over-counting is to compare the acceleration against a maximum vertical displacement threshold [20]. It is noted that peak-detection algorithms are characterized by low complexity; however, they are inefficient for various operation modes in which the inertial sensor changes pose during implementation.

However, the adoption of a similar approach to acquire the number of cycles performed by wheel-based vehicles would be much more effective in comparison to typical PDR applications. Such premise resides on the constrained rolling motion of wheels as opposed to the stepping nature of human motion. Hence, the acquired signal would be more uniform for the case of wheel-mounted accelerometers. Furthermore, for PDR applications, accelerometers are carried freely, which complicates the algorithm required to detect the stepping motion; however, for the AWOK system, an accelerometer is physically constrained to the wheel, which makes the acquired signal predictable. Hence, the AWOK signal is relatively easier to filter and process.

## 3. Background

This section covers the scientific background behind the proposed AWOK system, including the derivation of the mathematical model that describes the acquired acceleration signal from a radially wheel-mounted accelerometer. Additionally, the mathematical model provides the basis to construct the AWOK algorithm that is used to derive the wheel-travelled distance from the measurements of the radially wheel-mounted accelerometer.

Nevertheless, some assumptions are made upon which the mathematical model is derived. Initially, the inertial sensors assembly utilized for the AWOK system is preferable to be a full triaxial low-cost MEMS-based IMU. However, the AWOK system would render fully functional with just one radially mounted accelerometer. Furthermore, it is assumed that slipping or skidding conditions are not considered for the mathematical model, and accordingly for the preliminary phase of the AWOK algorithm.

Given an accelerometer, which is mounted with its sensitive axis aligned with the radial direction of a wheel of the wheel-based vehicle, and the wheel-based vehicle is stationary, the accelerometer should read out a component of the acceleration due to gravity (g) equivalent to the cosine of the initial inclination angle (θ) of the accelerometer’s sensitive axis, with respect to the direction of local gravity as given by Equation (1).

(1) $$a_{r}^{static}=g.\cos\theta_{i}$$

Nevertheless, if the wheel starts spinning about its centre without moving, the accelerometer should read a varying value of acceleration that follows the Equation (2), in which the inclination angle (θ) varies within the interval [0,2π]. Consequently, the relation expressed in Equation (2) can be reformulated as shown by Equation (3). It is noticeable that the spin component of acceleration would have cyclic behaviour with an angular frequency ($\omega_{s}$), which depends on the spinning speed of the wheel. Besides, the initial static component can then be considered as constant value, which can be represented as a phase shift (ϕ) to the cyclic behaviour of the spinning wheel accelerometer measurements. Consequently, the overall dynamic acceleration component can be rewritten as shown by Equation (4).

(2) $$a_{r}=a_{r}^{spin}\pm a_{r}^{static}=a_{r}^{spin}\pm g.\cos\left(\theta_{i}\right)$$

(3) $$a_{r}^{spin}=g.\cos\left(\omega_{s}t\right)$$

(4) $$a_{r}=g.\cos\left(\omega_{s}t\pm \phi \right)$$

In a more practical sense, a wheel-based vehicle typically rolls along its direction of motion, which introduces another component of radial acceleration. The radially mounted accelerometer would read out the additional acceleration due to the motion of the accelerometer with respect to the center of the wheel at the spinning angular rate ($\omega_{s}$), which in the physical sense is equivalent to the number of cycles per unit of time. Hence, the actual acceleration measured via the radially mounted accelerometer can be written as shown in Equation (5).

(5) $$a_{r}=g.\cos\left(\omega_{s}t\pm \phi \right)+\delta a_{r}$$

Whereas the additional radial component can be written as shown in Equation (6). In Equation (6), the radial acceleration component is equivalent to the product of the radius ($R_{e}$) at which the accelerometer is mounted on the wheel and the squared angular rate ($\omega_{s}$) of the spinning wheel.

(6) $$\delta a_{r}=R_{e}.\omega_{s}^{2}$$

Hence, the final readout of an accelerometer, which is radially mounted to a vehicle, can be given by Equation (7).

(7) $$a_{r}=g.\cos\left(\omega_{s}t\pm \phi \right)+R_{e}.\omega_{s}^{2}$$

## 4. AWOK System Setup

### 4.1. Design Parameters

#### 4.1.1. Accelerometer Input Range

As shown by Equation (7), the AWOK radially mounted accelerometer reading could be contaminated with an additional readout due to an additional radial acceleration component. The radial acceleration component is caused due to an eccentricity ($R_{e}$) in mounting the AWOK IMU to the wheel, where the centroid of the IMU does not coincide with the wheel center. Hence, by using the formula shown in Equation (6), that radial acceleration component increases quadratically with the angular rate of the wheel (ω). Consequently, the superimposed radial acceleration trend can be computed using Equation (6). Nonetheless, it can be beneficial to have a representation of the angular acceleration of the wheels, from which the vehicle speed can be derived. Such information can be considered redundant, and it can be implemented within the estimation algorithm by which the navigation states are acquired.

However, having a radial acceleration trend implies that the AWOK accelerometers, to be mounted on the wheels, should have a substantially high input range. The input range ($I_{a}$), in g, of the radially mounted accelerometer in the AWOK assembly can be identified using Equation (8).

(8) $$I_{a}=\frac{R_{e}}{g}.\frac{v_{max}^{2}}{R_{w}^{2}}$$

where ($I_{a}$) is the maximum input range for the radially mounted accelerometer of the AWOK assembly, ($R_{e}$) is the mounting radial eccentricity of the AWOK assembly centroid with respect to the wheel center, ($v_{max}$) is the maximum wheel-based velocity, and ($R_{w}$) is the overall wheel radius to which the AWOK assembly is mounted. To clarify, let us consider a standard sedan car that has a top speed ($v_{max}$) of 220 km/h, and a wheel rim diameter of 16 inches, and as an aspect ratio of 60% and wheel width of 205 mm; hence, a total wheel radius of 32.6 cm. Besides, let us assume a mounting radial eccentricity ($R_{e}$) of 1cm. Consequently, the maximum input range ($I_{a}$) for the radially mounted accelerometer of the AWOK assembly would be 10.64 g, which is within the typical specifications of most low-cost MEMS-based accelerometers.

#### 4.1.2. Accelerometer Bandwidth

A wheel-based vehicle depends on the rolling of its wheels to translate. As demonstrated earlier, the rolling of the wheels is a cyclical behaviour. Such cyclical behaviour can be modelled as a sinusoidal wave, whose period is variable as a function of the vehicle speed. To clarify, a vehicle moving at a slower speed would cause its wheel to complete a whole cycle in a longer period in comparison to moving at a higher speed. There exists a direct mathematical relationship between the period of a wheel cycle and the vehicle speed, which is given by Equation (9).

(9) $$T=\frac{D_{wheel}}{v_{vehicle}}=\frac{2\pi R_{w}}{v_{vehicle}}$$

where T is the periodic time for a wheel to complete one cycle. $D_{wheel}$ is the distance covered by a whole wheel cycle, and $R_{w}$ is the effective wheel radius. $v_{vehicle}$ is the linear velocity of the vehicle, where it can also be defined as the distance covered by the vehicle per unit time. Alternatively, the frequency of a rolling wheel ($f_{w}$) can be determined by reciprocating the periodic time (T), which is given by Equation (10).

(10) $$f_{w}=\frac{1}{T}=\frac{v_{vehicle}}{2\pi R_{w}}$$

Consequently, as a vehicle moves at higher speeds, the period of a complete wheel cycle would grow shorter. Ergo, the frequency required to capture such high dynamics would increase in return.

Theoretically, to capture such frequency, the accelerometer to be used for capturing the dynamics encountered by the AWOK system requires a bandwidth of at least twice the maximum frequency encountered due to the wheel motion. However, practically, the accelerometer bandwidth should be within five to ten times the maximum frequency encountered by the radially mounted accelerometer due to the wheel motion [22]. Hence, the lower threshold of the accelerometer bandwidth ($BW_{a}$) can be defined by Equation (11).

(11) $$BW_{a}=5.f_{w}=5.\frac{v_{vehicle}}{2\pi R_{w}}$$

For instance, if a car with a wheel radius of 32.62 cm moves at a constant speed of 100 km/h along a straight line, the complete wheel cycle would take 73.8 ms, which means a frequency of 13.6 Hz. Therefore, to capture such frequency, the radially mounted accelerometer to be mounted within the AWOK system should have a bandwidth of at least 68 Hz.

To conclude, the accelerometer bandwidth is a major design parameter for the AWOK system, to determine the maximum speed at which the AWOK system can perform, its suitability to the application, and the overall cost of the system as the accelerometer cost would increase with the increase of its quality. However, it is noted that most of the commercially available consumer-grade MEMS-based accelerometers would have a bandwidth of at least 200 Hz, which is sufficient to cover the navigation applications of regular passenger cars, which could reach a maximum speed up to 200 km/h. Such MEMS-based accelerometers can be readily found on the market at a price range within few U.S. dollars.

#### 4.1.3. Eccentricity Radius

Another obvious design parameter for the AWOK system is the radial mounting eccentricity between the AWOK-system accelerometer and the center of the wheel to which it is mounted. As shown earlier in Equation (7), the magnitude of the radial eccentricity increases the magnitude of the accelerometer readout, which is an erroneous signal component. Hence, it is crucial that the radial eccentricity is set to be zero. However, for the practical limitation of mounting the accelerometer to the wheel with a zero-value eccentricity, it should be taken into consideration that there should be a calibration procedure to identify the actual radial eccentricity value.

The calibration procedure is a simple procedure in which the wheel is rotated at a well-known angular rate while the vehicle is stationary. Afterwards, using the relation defined earlier in Equation (6), the radial eccentricity can be easily computed, as the accelerometer readout component is being logged through the calibration test period.

#### 4.1.4. Wheel Shape and Dimensions

As mentioned earlier, the AWOK system identifies the number of complete cycles covered by the wheels of a wheel-based vehicle. Nonetheless, to determine the overall distance covered by the vehicle, it is crucial to know the distance covered by the wheel per cycle. Hence, the wheel shape should be taken into consideration as a design parameter, as it would affect the distance covered by the said wheel.

**Figure 1:**
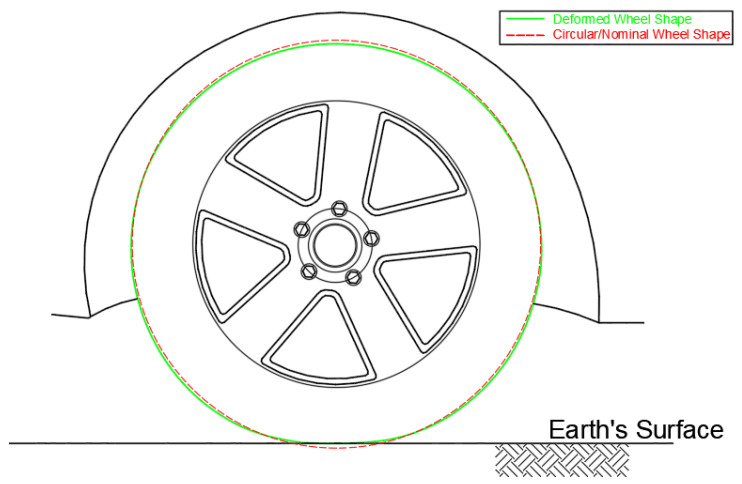
A schematic of the deformed wheel shape in comparison to the abstract assumption of circular wheel shape.

The wheel shape is always assumed to be circular in shape. However, in practice, it is common to find the wheel of the vehicle deformed under the impact of the vehicle’s weight, surrounding temperature, and tire air pressure or tire inflation level. Figure 1 shows a schematic of the deformed wheel shape in comparison to the abstract assumption of circular wheel shape. Such a deformed shape of the wheel would not conform to the assumptions made about the wheel. Consequently, it is important to account for such deformation.

There are several approaches by which one can calibrate for the deformed shape of the wheel. Nonetheless, for simplicity, the deformed wheel shape can be modelled as an alternate circular wheel shape with an effective radius. To specify the alternate circular wheel shape, it is assumed that the distance covered by the deformed wheel must be equal to the distance covered by the effective circular wheel shape. Consequently, the circumference of the deformed wheel shape is measured precisely, from which the effective wheel radius is deduced. However, the circumference of the deformed wheel shape should suffice, as the effective radius would render insignificant.

Consequently, the effective radius of the wheel to which the AWOK accelerometer is mounted is an extremely important aspect of the AWOK operation efficiency. To determine the effective wheel circumference, the wheel perimeter is measured precisely prior to motion. However, to maintain a sustainable algorithm that is not susceptible to errors because of varying wheel shape, there should be a more convenient approach that can provide a precise wheel circumference prior to each operation mission of the AWOK system, which is intended for future work.

### 4.2. System Design

For the initial prototype, the AWOK system is designed as an attachment that could be mounted to any wheel through bolts or screws, which ensures that the AWOK system performs its function, and as an effective means by which the AWOK system can be used for navigation of any wheel-based. At the same time, the main characteristic of the AWOK system is its capability to handle a wide range of dynamics. Besides, the AWOK system is designed in such a way to facilitate the testing and proof of concept process.

**Figure 2:**
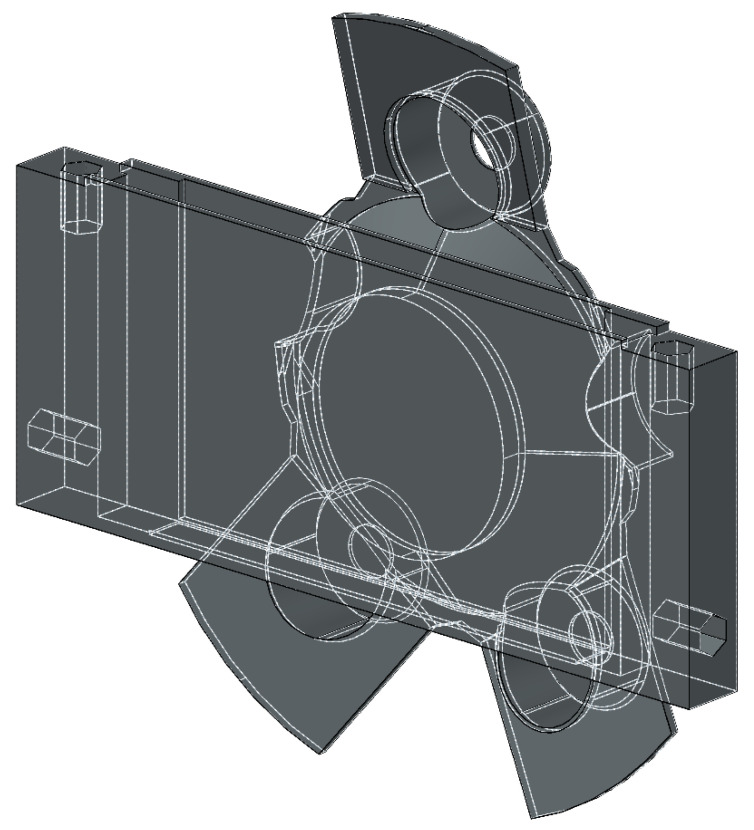
A schematic three-dimensional (3D) view for the design of the Accelerometer-based Wheel Odometer for Kinematics determination (AWOK) attachment.

For simplicity, the AWOK system was designed to be in the form of a Fused Deposition Modeling (FDM) 3D printed elastic attachment. The AWOK attachment was printed using tetra-polyurethane (TPU) material. The AWOK attachment could be attached to a car wheel using its typical wheel mounting bolts. The 3D printed part contains a compartment that enables installing an IMU within said compartment such that the radial eccentricity between the centroid of the IMU and wheel center is minimal. Figure 2 shows a schematic 3D view for the design of the AWOK attachment. Although the schematic figures from 2 to 4 show the AWOK attachment for a regular car, as a representative for wheel-based vehicles; however, the attachment can be easily modified to fit any wheel-based vehicle. The modification of the AWOK attachment can be easily achieved by making use of the high customizability of 3D printing and/or CNC machining.

In a typical AWOK system design, the system would include a standalone triaxial IMU, which can interface with a data logger and a processing unit. Hence, the AWOK data processing algorithm could be implemented directly, or the data can be processed in post-mission mode.

Nonetheless, the AWOK hardware assembly mount could take any other form, in which an accelerometer is mounted to the wheel of a wheel-based vehicle in a way that minimizes the radial eccentricity of the accelerometer’s centroid with respect to the center of the wheel. Hence, the AWOK assembly can take the form of an attachment that is mounted externally to the wheels of a wheel-based vehicle, as explained earlier for the initial prototype. Furthermore, The AWOK hardware assembly can take be integrated seamlessly within the vehicle wheel design to have a compact design.

### 4.3. Algorithm Description

The aim of this algorithm is to process the AWOK accelerometer dataset as acquired from the onboard radial accelerometer, which was mounted on the wheel-based vehicle, as described earlier in Section 4.2. Throughout the algorithm description, a portion of the acquired AWOK radial accelerometer data is used to demonstrate the steps of the AWOK algorithm. The acquired AWOK radial accelerometer was mounted on a car wheel. However, the rest of the results are going to be discussed in detail in Section 5.3, in which the experimental validation of the AWOK system is covered.

**Figure 3:**
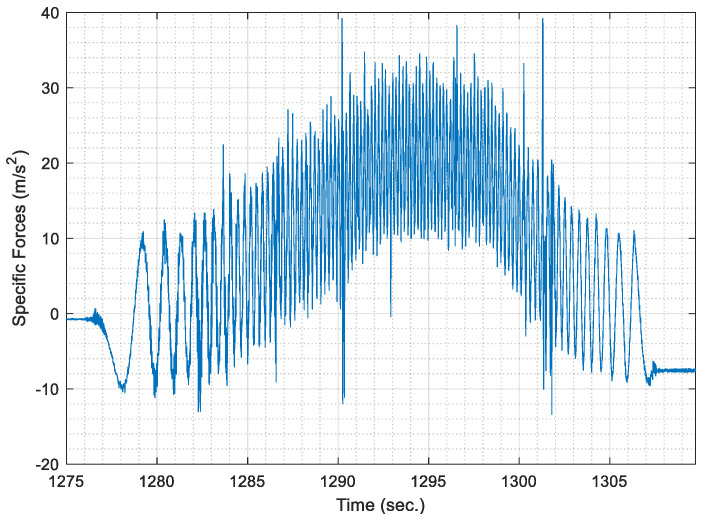
Raw specific forces time series for a sample of data as acquired from the radially mounted accelerometer.

Figure 3 shows the raw specific forces time series for a sample of data as acquired from the radially mounted accelerometer. It is noted that the shown sample was acquired during a segment whose length was nearly 200 m, with a maximum speed of 40 km/h.

It can be noticed from Figure 3 the cyclical behaviour of the specific forces is repeated whenever the wheel completes a complete cycle. It is noticeable that the wheel cycles are much more discernable than those cycles, which occur in the datasets acquired for PDR applications. Besides, Figure 3 shows an underlying trend in the form of a radial acceleration component that creates a curvature superimposed to the cyclical behaviour of the signal.

#### 4.3.1. Low-Pass Filter

The objective of this step is to remove the high-frequency noise within the acquired AWOK signal, which would facilitate the peak and valley detection of the signal, and ensure perfect distinction among peaks and valleys, which is discussed hereafter.

**Figure 4:**
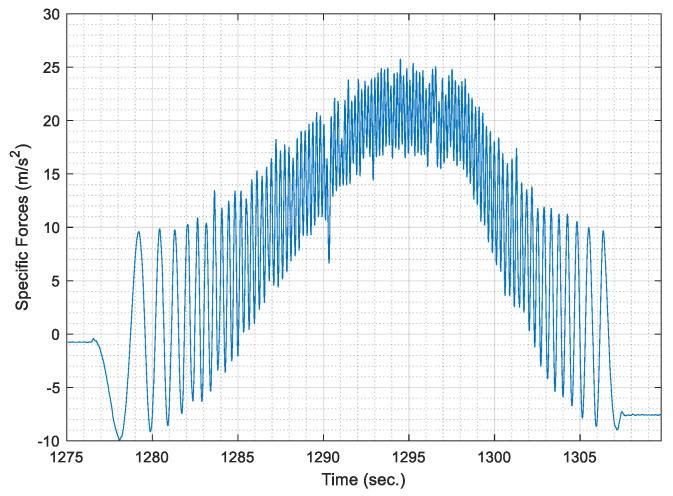
Smoothed AWOK accelerometer signal for a portion of the acquired data.

Hence, the acquired raw signal of specific forces is smoothed using a low pass filter. For simplicity, the low pass filter is implemented through overlapping moving averaging over a sampling window, which represents a fraction of the data rate. The fraction is taken as 10% of the maximum data rate for the acquired accelerometer signal, as it was found the most effective fraction to remove high-frequency noise in the signal. Moreover, the most effective overlapping moving average filter order was chosen as a third order filter, where the acquired AWOK signal is smoothed three consecutive times with the same window size. Figure 4 shows the smoothed AWOK accelerometer signal for a portion of the acquired data to show the effect of the data smoothing process.

#### 4.3.2. AWOK Signal Normalization

As per Section 3, it was derived that the AWOK radially mounted accelerometer should read out a sinusoid whose amplitude is equal to the acceleration due to gravity (g). However, to simplify the data processing and distance computations, the acquired AWOK signal is normalized such that the values of a typical AWOK signal would vary within the range [0,1].

To acquire the AWOK normalized signal ($x_{norm}$), Equation (12) is implemented. Whereas the raw AWOK accelerations signal ($x_{raw}$) as measured in $m/s^{2}$ is divided by the magnitude of the acceleration due to gravity (g). Consequently, the resulting signal is added to a value of 1, such that the values would vary within the interval [0,2]. Finally, the signal is multiplied by a factor of 0.5 to achieve the required normalization over the interval [0,1].

(12) $$x_{norm}=\frac{1}{2}\left(\frac{x_{raw}}{g}+1\right)$$

**Figure 5:**
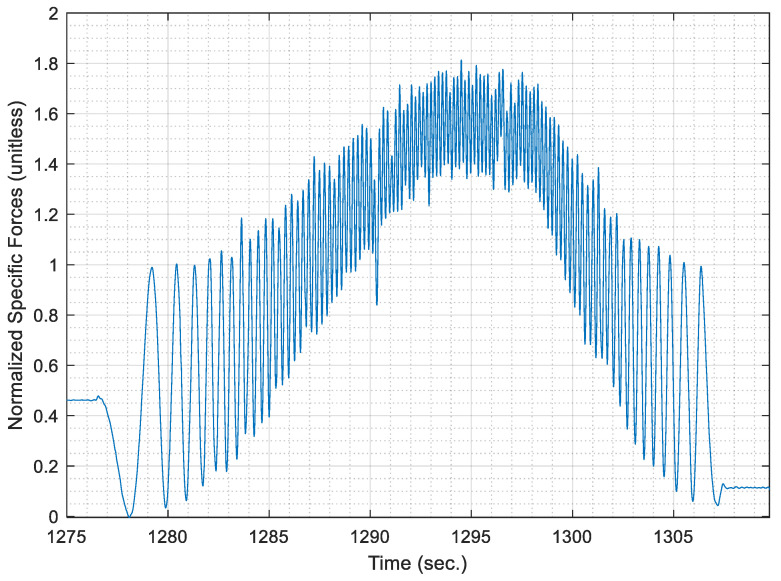
Normalized AWOK accelerometer signal for a portion of the acquired data.

The results of the AWOK signal normalization process can be demonstrated by Figure 5, which shows the same portion of the AWOK signal as depicted by Figure 4. However, in Figure 5, the AWOK signal is normalized over the interval [0,1].

#### 4.3.3. AWOK Signal Segmentation

The aim of this step is to sub-divide the trajectory into moving and stopping segments, such that the data processing scheme would be applied to the moving segments of the trajectory. Hence, the on-track distance can be acquired accurately. To achieve sub-division of AWOK accelerometer signal into motion and rest segment, a sub-algorithm is devised to determine the points at which the wheel-based platform starts and ends its motion.

Initially, the standard deviation of the acquired AWOK signal is computed at non-overlapping windows, which would be of the same size as the AWOK accelerometer data rate, as depicted by Equation (13). To clarify, if the AWOK accelerometer operates at 100 Hz, then the standard deviations are computed at windows of width 100 samples from the acquired signal.

(13) $$\sigma_{w}=\sqrt{\frac{1}{w}\overset{w}{\underset{i=1}{\sum }}\left(x_{i}-{\bar{x}}_{w}\right)},\;\;w=f_{s}$$

where ($\sigma_{w}$) is the standard deviation of the normalized AWOK signal over the window width (w) samples, which is equivalent to the accelerometer data rate ($f_{s}$). ($x_{i}$) is a normalized AWOK signal sample, and ($x_{w}$) is the mean of the normalized AWOK signal over the window width (w).

Whenever the standard deviation of the signal window is low, then the vehicle is static. On the other hand, if the standard deviation of the signal window increases, then this would mean that the accelerometer is reading high fluctuations within the signal. Such high fluctuations can only be caused by the cyclic motion of the wheel to which it is mounted.

The threshold by which the standard deviations classifies the motion state of the vehicle can be easily defined as per the known nature of the signal and what readout to expect when the wheel is moving. To specify, it can be reliably assumed that the acquired AWOK signal would represent a wide-sense stationary signal whose statistical properties would remain the same over prolonged periods of time [23].

**Figure 6:**
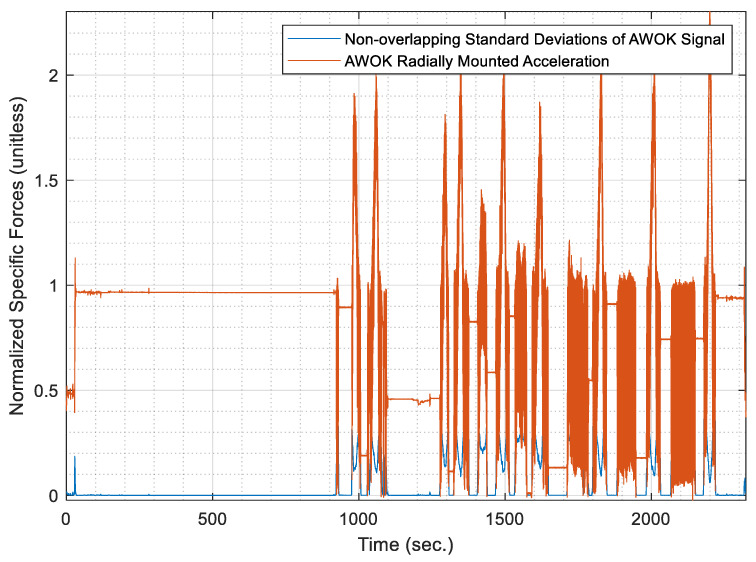
Normalized AWOK signal for a dataset overlaid with non-overlapping standard deviations.

Evidently, the AWOK accelerometer readout would fluctuate at least over the range [−g,g]. Nevertheless, if one considers the normalized version of the signal such that the fluctuations of the signal vary between [0,1], as performed in the previous step of the algorithm; hence, the standard deviation of said signal could be expected to maintain a standard deviation of 0.5. Figure 6 shows the normalized AWOK signal for a dataset that was acquired using a car that contains different static and dynamic portions to demonstrate the signal segmentation process. Within Figure 6, the non-overlapping standard deviations are overlaid on top of the normalized AWOK signal.

However, to account for any data artifacts, the threshold to classify motion and stationary segments is taken as 0.25, whereas a static wheel is expected to register a signal stand deviation, which is equivalent to the noise level within the static signal of the utilized accelerometer. Consequently, it is assumed that the standard deviation of the accelerometer signal when static would be within the order of magnitude of the white noise component within the sensor. Therefore, there would be a definite distinction between portions of static and dynamic readout of the AWOK accelerometer.

**Figure 7:**
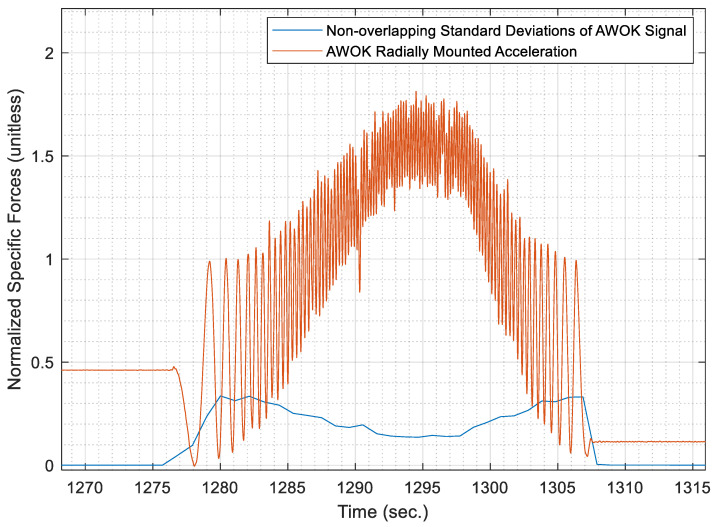
Overlay of the original AWOK signal over the computed standard deviations for a portion of the signal at which maximum speed was 40 km/h.

Otherwise, the portions of the signal that would register a normalized standard deviation less than 0.25 and more than that of the white noise of the sensor can be considered as artifacts. Such artifacts plausibly occur due to vibrations or due to loss of signal components because of inadequate accelerometer bandwidth or input range. Consequently, it is extremely important to choose an AWOK accelerometer that is suited to the expected dynamics, as discussed earlier in Section 4.1, to avoid faulty identification of the vehicle motion status, and avoid loss of distance information. This problem can be clarified by Figure 7, which shows the acquired standard deviations of the AWOK signal over windows of 100 samples for the AWOK signal as mounted for a car wheel moving at a maximum speed of 40 km/h preceded by a short static period. Besides, Figure 7 overlays the original AWOK signal over the acquired standard deviations.

It can be noticed that the predefined threshold of 0.25 on the normalized standard deviation would lead to classify a portion of the AWOK signal wrongfully as a static portion, whereas it is a portion of the motion period. Such an effect occurred due to the inadequacy of the used accelerometer to capture the velocity at which the car was moving. Nevertheless, to get around such a problem, the threshold was lowered to be 0.05 out of the normalized AWOK signal. However, in a typical case, in which the AWOK accelerometer is adequate for the expected dynamics, the threshold of 0.25 would suffice.

To sum up, the binary classification index (κ) of the normalized AWOK signal into motion and stationary portions can be computed by implementing Equation (14). For the classification index (κ), a value of one is assigned to portions of the signal in which the vehicle was in motion, and a value of zero is assigned to portions in which the vehicle was stationary.

(14) $$\kappa =\{\begin{array}{c} 1,\;|\;\sigma_{w}>threshold\\ 0,\;|\;\sigma_{w}\leq \;threshold \end{array},\;\;\;\;\;threshold=0.25$$

Consequently, the acquired AWOK signal is classified into portions of 1 s, which are flagged as either static or dynamic. The static periods are discarded for further processing through the AWOK algorithm; however, one can make use of such periods as zero velocity updates (ZUPT) within a typical Kalman filter implementation. Whereas, for each dynamic segment within the acquired AWOK signal, the rest of the AWOK algorithm would be applied to acquire the covered distance.

#### 4.3.4. Detect Peaks and Valleys

This phase of the algorithm is considered the pinnacle, whose objective is to determine the number of complete cycles covered by the wheel to which the AWOK assembly is mounted. Consequently, the peaks and valleys need to be identified within the cyclical signal where the period separating every two successive peaks or valleys mark a complete cycle of the wheel.

To identify the peaks within the normalized AWOK signal, the signal is differentiated with respect to time. Afterwards, the points at which the signal time gradient equates to zero are marked as local maxima and local minima of the signal, which correspond to either a peak or a valley, which satisfy the condition shown by Equation (15).

(15) $$\dot{x}=\frac{\partial x}{\partial t}=0$$

where ($\dot{x}$) is the time derivative of the normalized AWOK acceleration signal. The detected signal peaks are then filtered to remove noisy peaks, which occur due to imperfections in the signal, such as vibrational and impact effects due to car motion, improper filtering errors. The peaks filtration phase takes place by satisfying a simple arithmetic condition, as shown by Equation (16), in which the detected peaks must surpass half the mean value of the magnitudes of the peaks.

(16) $$∥x_{peak}∥>\frac{1}{2n}.\overset{n}{\underset{i=1}{\sum }}{\left(x_{peak}\right)}_{i}$$

**Figure 8:**
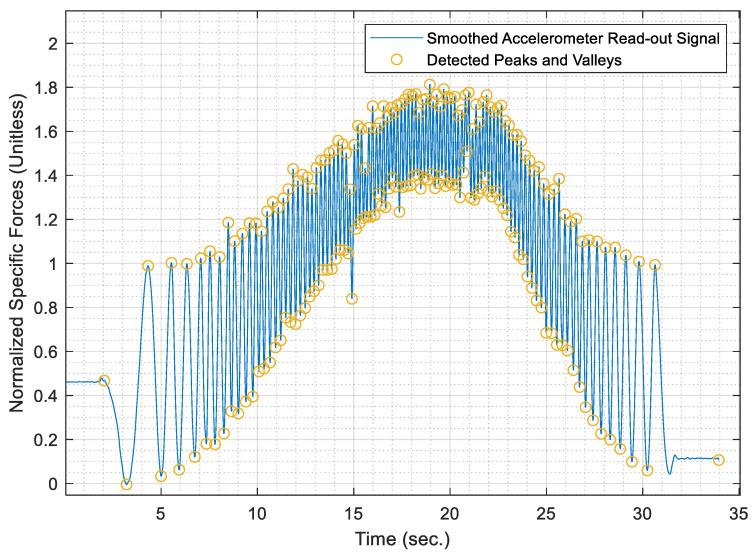
A portion of the AWOK signal at which the vehicle reached a maximum speed of 40 km/h superimposed with the detected Peaks and Valleys.

Therefore, the data peaks and valleys within the smoothed accelerometer signal are identified and superimposed over the smoothed signal, which is depicted in Figure 8 for a portion of the AWOK signal at which the vehicle reached a maximum speed of 40 km/h.

#### 4.3.5. Compute Phase Shift Values

Once the number of complete cycles is determined, as explained in the previous step, it is still required to determine the partial cycles the wheel covers at the start and the end of each motion segment. These partial cycles are referred to hereafter as phase shift. Whereas, phase shift typically represents a fraction of a complete cycle in a typical sinusoid, which is depicted by the model discussed earlier in Section 3, within Equation (7), as the parameter (ϕ).

**Figure 9:**
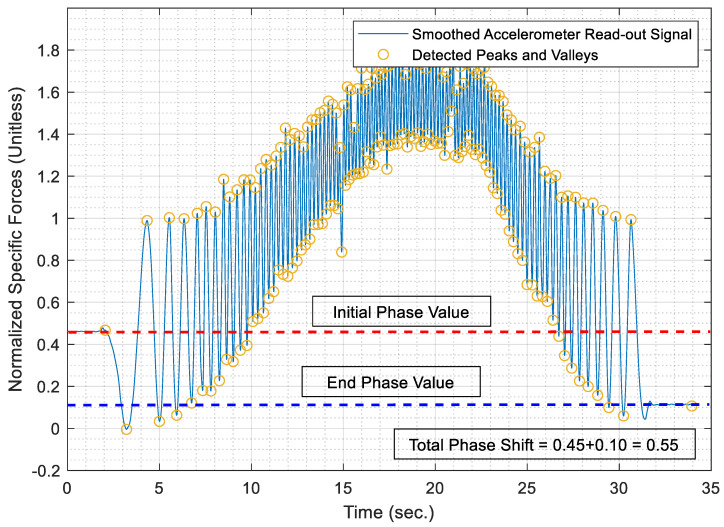
The phase shift values at the start and end of a motion segment within the acquired AWOK signal.

Consequently, the phase shift value can be determined from the normalized AWOK signal. The total phase shift value represents the phase shift value at the start of the motion segment signal, in addition to the phase shift value at the end of the motion signal. Whereby, the phase shift value is computed from the ratio between the magnitude at which the motion segment started and ended to the maximum magnitude expected from the AWOK signal. It is noted that the acquired AWOK signal should typically experience a maximum magnitude of (g). Figure 9 shows the phase shift values at the start and end of a motion segment within the acquired AWOK signal.

Since the signal is normalized within the interval [0,1], then the normalized acceleration value at the start point of the motion segment time series, under study, would directly represent the start phase shift value. Similarly, the normalized acceleration value at the end of the motion segment would directly represent the end phase sift component. Hence, the total phase shift of said motion segment is computed by summing up the start and end phase shift values.

(17) $$\phi =\frac{x_{start}+x_{end}}{g}=\phi_{start}+\phi_{end}$$

where ($x_{start}$) represents the filtered acceleration value measured in ($m/s^{2}$) at the start of the motion segment within the AWOK signal. While ($x_{end}$) represents the filtered acceleration, value measured in ($m/s^{2}$) at the end of the motion segment within the AWOK signal. Consequently, ($\phi_{start}$) and ($\phi_{end}$) represent the phase shift values at the start and end of the AWOK motion segment, respectively.

#### 4.3.6. Complete Travelled Distance

Finally, the complete travelled distances can be computed by applying Equation (18). Simply, the total travelled distance within any given motion segment using the AWOK system can be computed by the product of the total number of cycles covered by the wheel and the circumference of the wheel to which the AWOK system is attached.

(18) $$D=\left(N_{p}+\phi \right).C_{w}=\left(N_{p}+\phi \right).\left(2\pi R_{w}\right)$$

where (D) is the complete travelled distance by the AWOK system through any given motion segment within the acquired data. ($N_{p}$) is the number of complete cycles covered by the wheel to which the AWOK system is attached, which is determined from the acquired number of peaks, as discussed in Section 4.3.4. ($C_{w}$) is the wheel circumference which can be measured physically from the wheel itself or computed given the effective radius value ($R_{w}$), as discussed in Section 4.1.4.

#### 4.3.7. AWOK Algorithm By-Products

As discussed earlier, the behaviour of the acquired radially mounted accelerometer signal is characterized by having a cyclical behaviour, which repeats over a range of periodic times per cycle. In a sense, the periodic times reflect the on-track speed of the wheel-based platform. Nonetheless, the periodic time values should vary in magnitude depending on the vehicle’s speed and acceleration.

Since the distance covered by the wheel during one cycle is a well-known value, then the vehicle’s speed and acceleration can be computed as well. Hence, the speed values are computed for each cycle completed by the wheel, as demonstrated by Equation (19).

(19) $$v_{c}=\frac{C_{w}}{T}=\frac{2\pi R_{w}}{T}$$

where ($v_{c}$) is the velocity of the vehicle at any given peak or valley along with the acquired AWOK acceleration time series. (T) is the time that takes the wheel to complete one cycle; in other words, (T) is the time between any two successive peaks or valleys along with the AWOK acceleration time series. Finally, ($C_{w}$) is the wheel circumference which can be measured physically from the wheel itself or computed given the effective radius value ($R_{w}$). It is noted the relation depicted by Equation (19) is the same as the one expressed earlier in Section 4.1.2., by Equation (9). Hence, the acceleration signal can be computed by numerically differentiating the acquired time values and with the well-known distance covered by the car wheel over one cycle. Consequently, the acquired acceleration and velocity values can be used as update values in Kalman filter implementation besides the distance value, which is acquired typically from the AWOK system.

Another by-product that can be computed is the angular velocity of the car wheel. The angular velocity of the car wheel can be computed by determining the periodic time taken by each cycle. Since each cycle represents a 360° rotation, then the angular speed of said cycle can be easily calculated by dividing the 360° by the time taken to complete the cycle.

The angular rate of the wheel can be beneficial in eliminating the low-frequency component error that occurs as an additional acceleration readout component in the AWOK accelerometer data due to the radial eccentricity of mounting the AWOK accelerometer to the wheel center. Whereas, by using Equation (6), the radial eccentricity value can be determined and can be averaged over time to have an online best estimate for the radial eccentricity without additional testing of the system.

**Figure 10:**
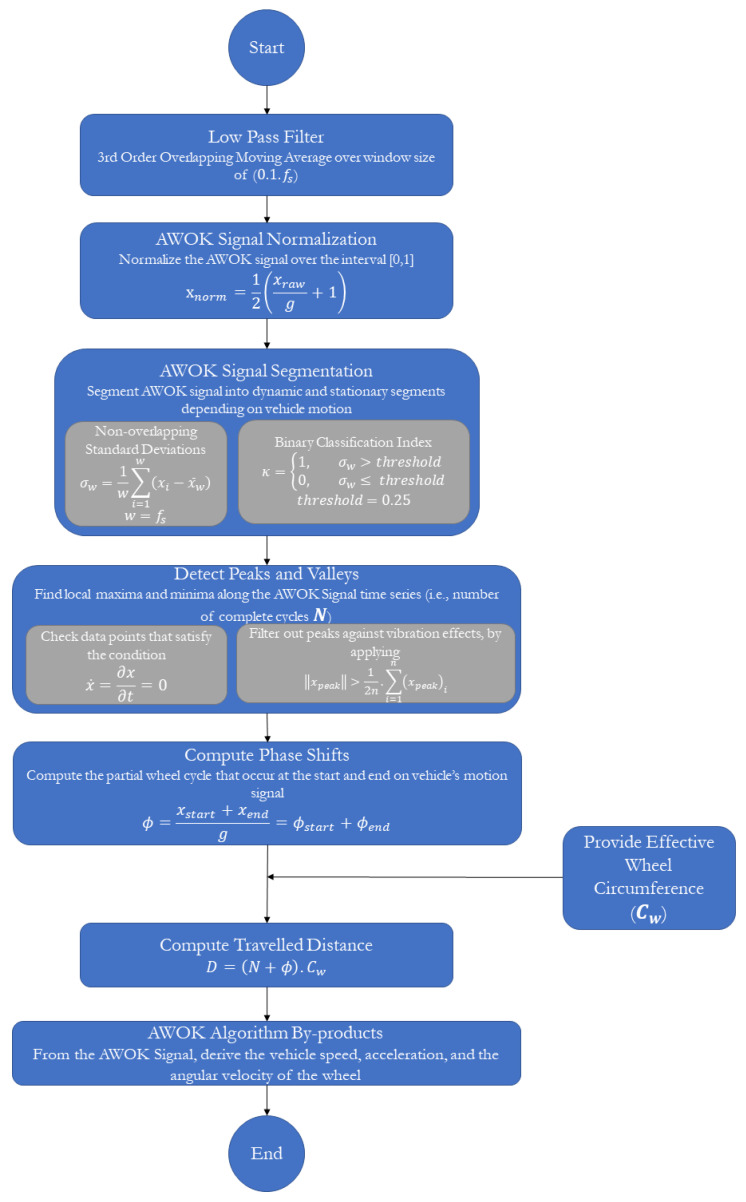
AWOK algorithm flow chart.

To conclude, Figure 10 shows a flow chart of the complete AWOK algorithm showing the main steps needed to acquire the covered distance by the wheel-based vehicle, along with the speed and acceleration of the said vehicle.

## 5. AWOK Controlled-Environment Experiment

This section covers the experimental validation of the AWOK system in a controlled environment. Whereby, an experiment was held to verify that the AWOK system, including the AWOK hardware assembly and algorithm, would yield stable and accurate distance results of a wheel-based vehicle. To specify, the objectives of this experiment is to provide a proof of concept for the AWOK system, validation, and proving that the AWOK system results are invariant to changes in dynamics.

### 5.1. Experimental Setup

#### 5.1.1. AWOK Hardware Assembly

Prior to performing the experimental validation of the AWOK system, a 3D design was made for the initial prototype of the AWOK assembly. The design was made for an AWOK assembly that can be mounted to the wheels of a Ford Fusion car whose production year is 2008. The design was performed by initially 3D modelling the Ford Fusion SE car wheel, with determining the exact dimensions of the wheel bolts, which were five bolts of M12 size.

For experimentation, a smartphone was used as a replacement for a standalone IMU to capture the radial acceleration readout component. Most of the commercially available smartphones contain triaxial accelerometers, which are sufficient for the AWOK system to function. The smartphone that was used was an LG X Power android smartphone. The on-board accelerometer is an LGE accelerometer sensor whose performance evaluation can be found in [24]. However, it is noted that the android app that was used for logging the acceleration data limited the maximum input range to 4 g, instead of 16 g. Additionally, the bandwidth of the on-board accelerometers was set to 100 Hz, which was sufficient to capture the dynamics expected within the AWOK system tests.

#### 5.1.2. Reference Data

To provide reference distance data against which the AWOK data could be measure, a Total Station was used to measure the distance covered by the car to which the AWOK assembly is attached. To use a total station, a direct line of the side should be kept between the total station and the target point. A Leica TCR08 was used to perform the distance measurements, which can acquire 3D positions with ±1 cm at a 95% confidence interval.

Consequently, a reflector prism was mounted on top of the car trunk via a magnetic plate. The total station would record the position of the car prior to its motion and records the position once more at the end of the test distance. Afterwards, the distance between the start and endpoints is computed if the car moved along a straight line during the test. It is noted that the car was moved along the distance while maintaining a perfectly still position of the steering wheel to ensure that the car is moving along a straight line.

**Figure 11:**
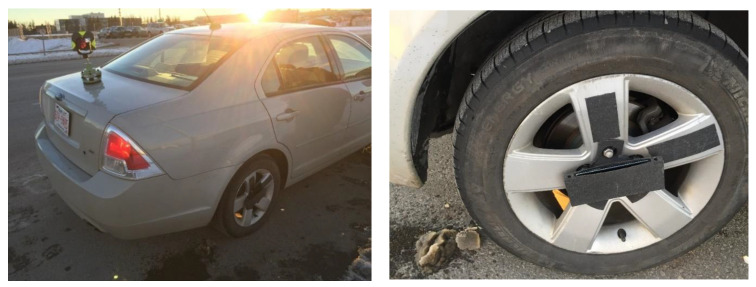
Complete AWOK experimental setup.

The car was assumed to be a rigid body such that it can be represented geometrically by a point. In such a case, the distance acquired from the total station (i.e., reference distance) is equivalent to that acquired distance from the AWOK data processing algorithm, neglecting the lever arm between the wheel, to which the AWOK hardware assembly was mounted, and the total station reflector prism. The complete experimental setup is shown in Figure 11.

#### 5.1.3. Effective Wheel Circumference Calibration

As mentioned earlier in Section 4.1.4, the effective wheel circumference is a crucial aspect upon which the accuracy of the AWOK system resides. Consequently, there had to be a method by which the effective wheel circumference had to be determined precisely up to ±0.001 m, to ensure high level of distance measurement accuracy. To clarify, as per the mathematical model expressed by Equation (18), the AWOK distance error is dependent upon the error in acquiring the complete number of cycles performed by the wheel and the error in measuring the effective wheel circumference.

Nonetheless, as explained earlier, the AWOK algorithm is efficient in determining the number of cycles performed by the wheel due to the special nature of the raw acceleration signal. It is noted that the total number of cycles performed by the wheel includes the number of complete wheel cycles, in addition to the partial cycle that occurs at the start and the end of the wheel motion, as explained earlier in Section 4.3.5. Hence, it is assumed that the capability of the AWOK algorithm in determining the number of cycles has an extremely high level of confidence, with low error, such that it would not affect the AWOK-system distance measurement.

Ergo, the main source of error expected to affect the AWOK-system distance measurement would be the effective wheel circumference measurement. Moreover, it is noted that the error in the effective wheel circumference is a systematic error, which would manifest as a scale factor, which would increase with the increase in the travelled distance by the vehicle. Consequently, to ensure the high performance of the AWOK system, the effective wheel circumference had to be calibrated and determined with extremely high accuracy.

Therefore, one of the acquired datasets within the experiment was used as a control set, in which the total number of cycles was determined using the AWOK algorithm. Moreover, the travelled distance was then acquired using the Total station with an accuracy of ±0.01 m. The Total Station distance as measured on the field was 193.867 m. Afterwards, the effective wheel circumference could be determined by dividing the total distance by the total number of wheel cycles. Furthermore, by implementing the law of variance propagation to the simple mathematical model, expressed earlier by Equation (18), the standard deviation of the computed wheel circumference could be determined, as depicted by Equation (20).

(20) $$\sigma_{C_{w}}^{2}=\frac{\sigma_{D}^{2}}{\left(N_{p}+\phi \right)}$$

where ($\sigma_{D}^{2}$) is the variance of the distance measured using the Total Station that is taken as 0.0001 $m^{2}$, ($N_{p}+\phi$) is the total number of cycles as acquired from the AWOK algorithm for the control dataset, which was 97.6793 cycles. ($\sigma_{C_{w}}^{2}$) is the variance of the computed wheel circumference. Therefore, the circumference of the car wheel was measured precisely to be used in distance evaluation within the AWOK algorithm. Whereas the car wheel circumference was 1.985 m with a standard deviation of ±0.001 m.

### 5.2. Experiment Description

**Figure 12:**
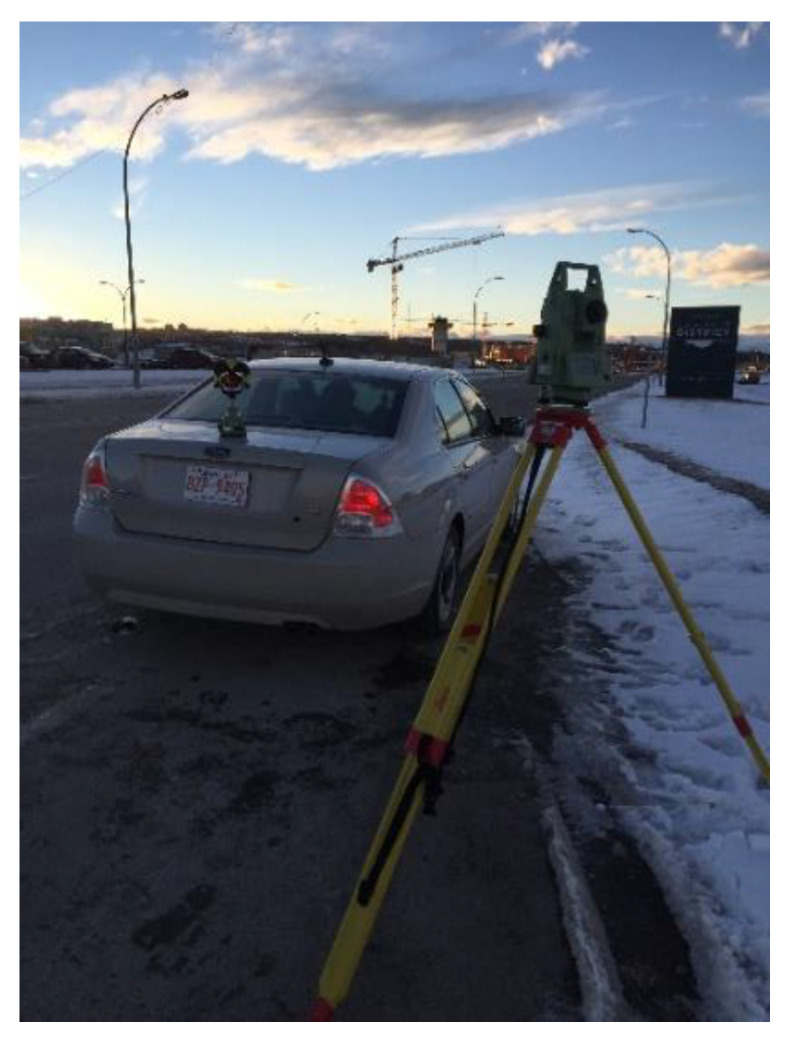
Vehicle as viewed from the perspective of total station user.

The experiment was held on campus within the University of Calgary. The trajectory on which the experiment was held was a straight segment of a total distance of about 190 m. The straight segment was marked by the road curb, and its straightness was checked via the total station. The AWOK system was used to acquire a test distance five consecutive times at 5, 10, 20, 30, and 40 km/h speeds. As stated earlier, the car was kept stationary at the start and end of each test distance, such that the total station could acquire the 3D position of the car. Figure 12 shows an image for the vehicle prior to one test distance, as viewed from the perspective of the total station user.

Nonetheless, the limited test distance and speeds can be attributed to the use of a total station to acquire the reference distance data. Besides, the test was repeated five consecutive times in an area with moderate traffic flow, which limited the stopping time and speed limit. Furthermore, the distance and speed were limited to ensure that the vehicle motion was on a straight line marked by the shoulder line of the road on which the tests were performed and to be able to keep the steering wheel still over the whole trajectory.

### 5.3. Results

**Figure 13:**
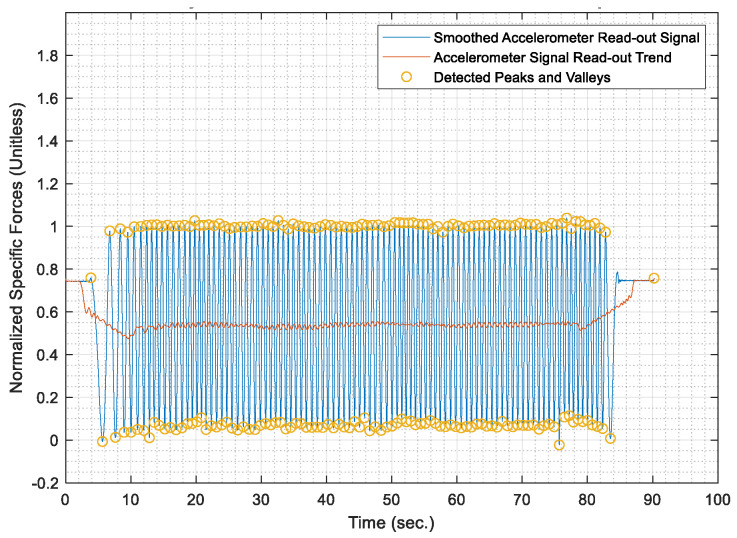
Processed AWOK radially-mounted accelerometer readout at maximum speed 5 km/h.

**Figure 14:**
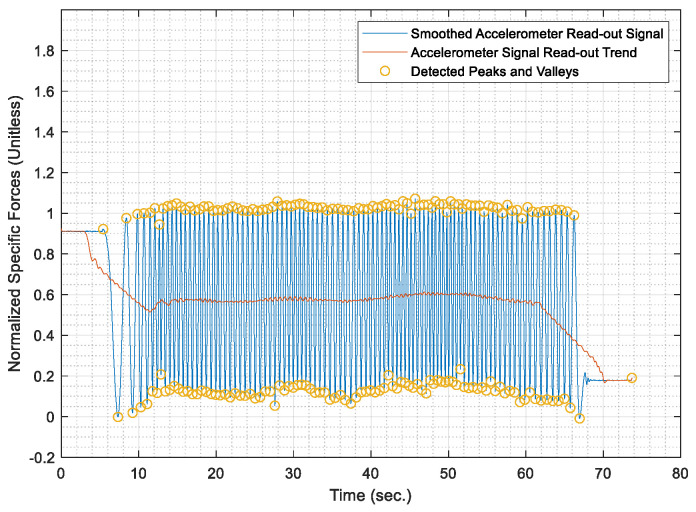
Processed AWOK radially-mounted accelerometer readout at maximum speed 10 km/h.

**Figure 15:**
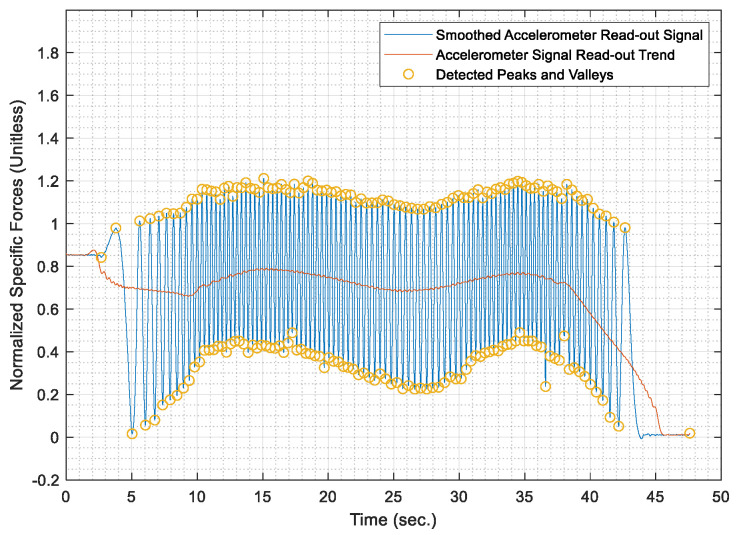
Processed AWOK radially-mounted accelerometer readout at maximum speed 20 km/h.

**Figure 16:**
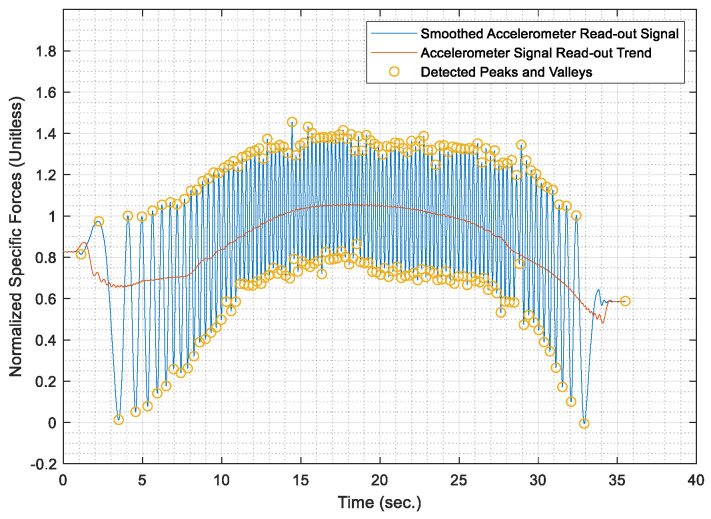
Processed AWOK radially-mounted accelerometer readout at maximum speed 30 km/h.

**Figure 17:**
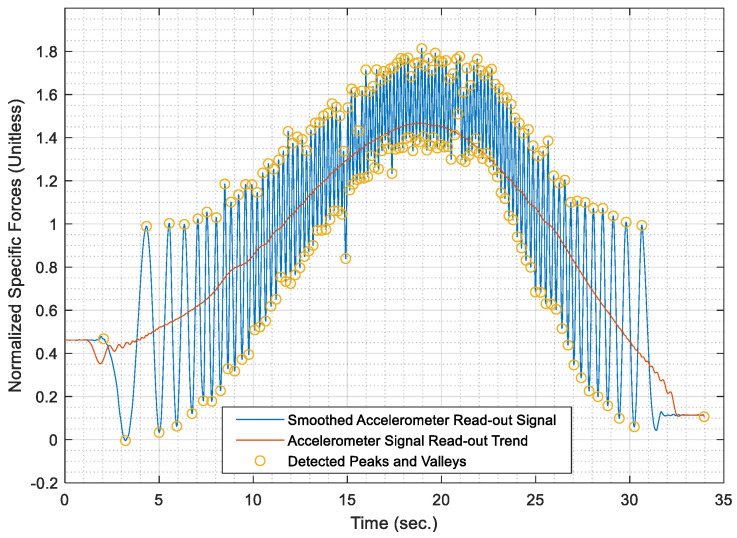
Processed AWOK radially-mounted accelerometer readout at maximum speed 40 km/h.

Once the test distances were acquired, the AWOK acceleration signals were run through the AWOK algorithm to get the distances covered by the car at the different test speeds and compare such distances against their corresponding values from the total station. Figures from Figure 13, Figure 14, Figure 15, Figure 16 and Figure 17 show the normalized acceleration signals as acquired from the AWOK system at speeds 5, 10, 20, 30, 40 km/h, respectively. Besides, figures from Figure 13, Figure 14, Figure 15, Figure 16 and Figure 17 show the trend of each acquired signal and the detect peaks and valleys within each acceleration signal.

Whereas Table 1 shows the results of comparing the AWOK-acquired distances against the reference total station distances, which show that the AWOK distances are precise in comparison to the reference total station regardless of the speed at which the vehicle was moving. Besides, it is noticeable that the distance errors are random and do not include any systematic trends. It is reported that the root mean square error (RMSE) of 0.042 m for the five-distance tests in which the AWOK system was tested.

### 5.4. Observations

By observing Figure 15, Figure 16 and Figure 17, there are trends that appear with the increase of velocity, which is an indication of radial mounting eccentricity, which makes the AWOK radial accelerometer read an additional acceleration component that manifests in the final acquired data as a long-term component within the signal.

As hinted earlier within Section 4.3.3, it can be observed from Figure 17 that there are some data loss within the magnitude of the acceleration signal, which occur with the increase of velocity because of using an accelerometer whose input range is limited to 4 g; which is not as per the specifications of the accelerometer mounted on the used smartphone. However, the limitation on the input range is set by the app that was used to acquire the data. Besides, data loss would occur because of filtering out high-frequency components of the signal using a third order filter. Nevertheless, such a problem would not occur if the AWOK system was mounted to the wheel with nearly zero radial eccentricity and using an accelerometer of higher input range and bandwidth.

The AWOK system output signal typically would include a low-frequency error component, which is either caused by radial eccentricity or the inherent stochastic error components within the accelerometers, such as drift ramp or bias instabilities. Nevertheless, it is noticeable from the acquired signal that such low-frequency error components have no effect on the detection of the peaks and valleys within the output signals. Such observations consolidate the idea that the AWOK system is insusceptible to low frequency error components in determining the covered distance by the vehicle.

Furthermore, the utilized algorithm provides the perfect distinction of peaks and valleys, which makes the AWOK system more efficient at detecting when the wheel has completed a complete cycle because the signal floor is relatively high in comparison to the PDR applications, in which a similar approach is used to detect pedestrian steps [14].

## 6. AWOK versus GNSS Experimental Validation

This section covers the experimental validation of the AWOK system distance and velocity measurement versus distances and velocity acquired using the Global Navigation Satellite System (GNSS) for a representative trajectory. An experiment was held to verify that the AWOK system, including the AWOK hardware assembly and algorithm, would yield stable and accurate distance results of a wheel-based vehicle in comparison to GNSS-acquired distance. In addition, this experiment tests the validity of the velocity results acquired from the AWOK system in comparison to the velocity, as provided by the electronic control unit (ECU) of the car, which is read out via an Onboard Diagnostic II (OBD2) adapter.

### 6.1. Experimental Setup Description

For this experiment, the AWOK hardware assembly, which was described thoroughly in Section 5.1.1, was used. It is noted that the same smartphone was used as a replacement for the triaxial accelerometer. As mentioned earlier, the smartphone implies some restrictions on the maximum speed at which the system could be tested. Consequently, the experiment was held such that the maximum speed was set to 40 km/h. However, the maximum vehicle speed could be increased without affecting the AWOK system performance, as explained earlier in Section 4.

Additionally, an OBD2 adapter was connected to the OBD2 port of the vehicle, and the velocity data was acquired from the vehicle at a data rate of 10 Hz. The velocity acquired for the vehicle speedometer was recorded and compared thereafter with the velocity acquired from the AWOK system, as shown afterwards.

#### 6.1.1. Reference Data

GNSS-acquired distances were used as reference data for the experiment. The GNSS was used in differential kinematic operation mode. Whereas, a Trimble R10 GNSS receiver was used as a base station occupying a well-known precise point, whose coordinates are known up to ±2 mm. At the same time, another Trimble R10 GNSS receiver was mounted on the vehicle. The data was processed post-mission using the Trimble Business Center software package. The GNSS data was 3-D coordinates points acquired along the trajectory covered by the vehicle at 1 Hz data rate. It is noted that the reported precision of the GNSS kinematic post-processing solution is ±1.5 cm for horizontal coordinates (i.e., easting and northing components) and ±2.5 cm for vertical coordinates. Hence, GNSS-acquired distances and velocities provide reliable and precise reference ground truth to test the AWOK system against.

#### 6.1.2. AWOK Wheel Circumference Calibration

**Figure 18:**
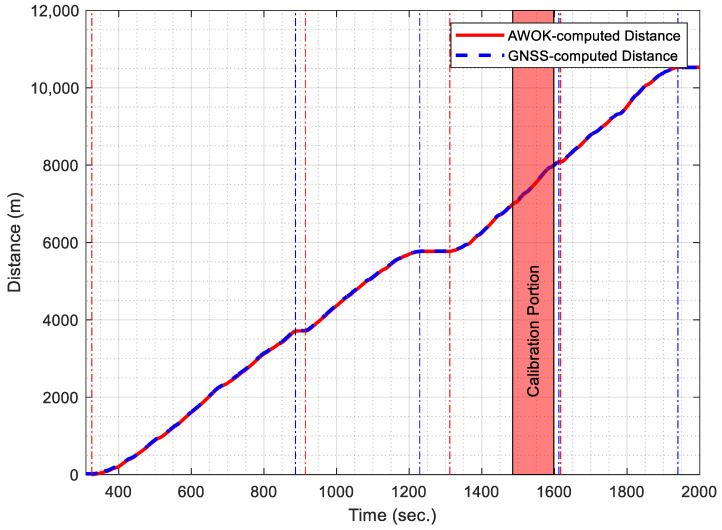
A comparison of the AWOK-acquired distance versus the GNSS-acquired distance.

To provide a calibrated wheel circumference to be used to compute total AWOK distance, a portion of the GNSS-acquired distance trajectory was used as a control set, in which the total number of cycles was determined using the AWOK algorithm. The GNSS-acquired control distance was taken as 763.397 m. Afterwards, the effective wheel circumference was determined by dividing the total distance by the total number of wheel cycles. Figure 18 shows the portion of the trajectory that was used as a control set overlaid as an area plot over the comparison between the AWOK acquired distance and the GNSS-acquired distance.

### 6.2. Experiment Description

**Figure 19:**
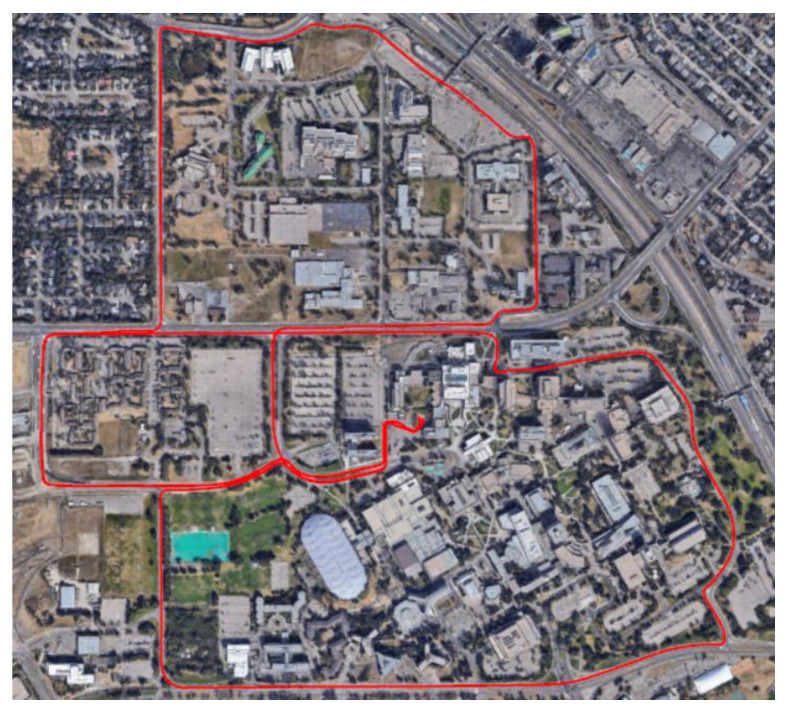
A map view of the test trajectory.

The experiment was held near the campus of the University of Calgary. The trajectory on which the experiment was held was a segment of a total distance of about 10 km. The AWOK system was used to acquire the distance and on-track velocity at a maximum vehicle speed of 40 km/h, as explained earlier. Figure 19 shows a map view of the test trajectory.

### 6.3. Results

Following the same procedure explained earlier and using the AWOK algorithm, the distance acquired using the AWOK system was computed and compared against the distance acquired from the GNSS solution. Figure 18 shows a comparison of the AWOK-acquired distance versus the GNSS-acquired distance.

**Figure 20:**
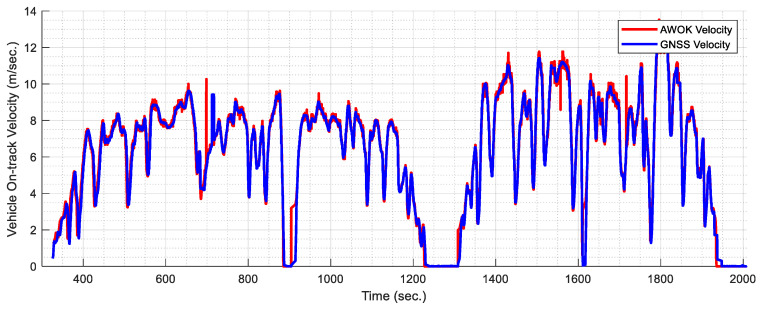
Comparison between the AWOK-acquired velocity and the corresponding on-track velocity as acquired from the GNSS coordinates.

As stated earlier, the AWOK system can provide the vehicle’s on-track velocity as a by-product of the AWOK algorithm, which can be beneficial to acquire a better navigation solution when integrated with additional sensors. Hence, the on-track velocity of the vehicle was computed using the AWOK algorithm and is compared to the on-track velocity computed using the GNSS coordinates of the vehicle along the trajectory. Figure 20 shows the comparison between the AWOK-acquired velocity and the corresponding on-track velocity as acquired from the GNSS coordinates.

Table 2 shows a summary of the results acquired from the validation of the AWOK system in comparison to the GNSS-acquired distances. Besides, Table 2 shows the RMSE of the on-track velocity acquired from the AWOK system with respect to the GNSS velocities. Furthermore, Table 2 shows the RMSE of the on-track velocity acquired from the vehicle’s OBD2 with respect to the GNSS velocities.

## 7. Discussion and Conclusions

From the held experiment, the results show that the AWOK system provides direct distance measurements instead of velocity measurements at high precision when compared to total station data. Such results were fortified by the results in comparison to differential GNSS solution for a full trajectory. Consequently, it can be stated that the AWOK system achieves a relative precision of 0.15% for distances. Hence, it can be concluded that the AWOK system provides a perfect replacement for typical wheel odometers. Typical wheel odometers are wheel encoders, which provide should provide a relative accuracy of at least ±4%, as stated in [25]. Besides, typical wheel odometers suffer from stochastic errors within their scale factor, which is modelled within any navigation filter for integrating the velocities acquired from said odometers as an additional state [26]. However, such stochastic errors are vastly diminished in the case of the AWOK system, as discussed earlier. Besides, the AWOK system provides such an alternative at a low cost. Besides, it has been shown, as per the experimental results, that the RMSE computed for the on-track velocity as acquired from the AWOK system is less than the RMSE for the on-track velocity from the OBD2 readout. Hence, the result is conclusive in terms of the advantage of the AWOK system over typical wheel encoders, which provide the read-out for the OBD2.

Furthermore, it can be deduced from the results that the AWOK data provides distances with no systematic trends of errors with the variation of velocity. Additionally, AWOK distances are insusceptible to inherent errors within the utilized low-cost MEMS accelerometers, as per the design of the data processing algorithm and the nature of the acquired signal.

However, the AWOK accelerometer input range and bandwidth should be increased to be able to capture higher dynamics because the AWOK experience minor data loss for the experiment at which the car moved at a maximum speed of 40 km/h. To generalize, the choice of an AWOK accelerometer must suit the intended vehicle and application to be navigated.

As discussed earlier in Section 4.3.3, the AWOK algorithm segments the acquired acceleration signal into stationary and dynamic portions. Hence, one could make use of such information to be implemented within any utilized navigation filter. Whereby, once the stationary segments are flagged within the acquired dataset, the stationarity of the vehicle can be used as zero velocity updates, as mentioned earlier.

Besides, the velocity can also be deduced precisely from the signal as a by-product from the AWOK algorithm, which would be extremely beneficial when implemented within a multisensory integration scheme through Kalman filter. In contrast, the AWOK velocity signal can provide additional update information, especially in GNSS-denied environments.

Since the AWOK algorithm can prove the acceleration as a by-product, such acceleration would be useful in a Kalman filter integration scheme. To clarify, the AWOK acceleration would be a better, more precise form of the actual on-track acceleration of the vehicle. At the same time, the AWOK acceleration is claimed to be more precise because of the nature of the AWOK signal, which provides cycle periodic times precisely regardless of the quality of the used accelerometer within the AWOK system. The highly precise periodic times would lead to a better estimate of the vehicle’s on-track acceleration, with low stochastic errors. Hence, the AWOK signal can be used in a Kalman filter, which includes a typical IMU, which is mounted on the vehicle to have a better estimate of the stochastic errors within the accelerometers of said IMU. Whereby, the AWOK accelerations can be fused with the raw IMU accelerations to perform, in some manner, an online calibration using cycle acceleration.

As stated earlier in Section 4.3.7, the AWOK algorithm provides the angular rate of the wheel as a by-product of the data analysis. Consequently, the derived wheel angular velocity can be used to calculate the radial acceleration component caused by the wheel rotation as it moves. Hence, if the AWOK system mount suffers from radial eccentricity, the radial eccentricity effect can be calibrated online, knowing the angular rate. At the same time, the additional radial eccentricity error can be removed from the acquired signal. Besides, the radial eccentricity distance can be computed in calibration mode prior to using the AWOK system for any navigation application.

To sum up, the AWOK system provides a more accurate replacement for wheel encoders that are utilized for navigation applications at a relatively low cost. Although similar approaches have been implemented previously in utilizing wheel-mounted accelerometers as odometers; however, the AWOK system can sustain extremely high dynamics in comparison to the similar systems introduced earlier. To clarify, similar wheel-mounted accelerometers were used for wheel odometry in low dynamics applications such as bicycles and wheelchairs. On the other hand, the AWOK system has been tested and validated for car navigation applications. Hence, the AWOK system can be readily implemented for autonomous car navigation systems, which are integral components for self-driving cars.

Nevertheless, vibrations have detrimental effects upon the measurements from the AWOK system. Vibrations are defined as any time data spike that registers an acceleration of a higher magnitude over a short period of time in comparison to succeeding or preceding acceleration values. In the context of the AWOK signal, a vibration is defined by an acceleration peak larger than twice the mean of previously computed peaks. Vibrations can be caused by the poor mechanical mounting of the AWOK System, imperfect wheel to vehicle mounting, or uneven terrain. Whereby such a cause would lead the AWOK accelerometer to register high acceleration magnitudes from what is expected. Hence, it is intended to address how to minimize the effects of vibrations on the final distance measurements of the AWOK system.

As stated earlier, the preliminary AWOK algorithm introduced within this research excluded the effects of skidding and slipping. Consequently, another aspect that should be covered within future work is the effect of wheel skidding and slipping over the AWOK system results. To clarify, the wheel skidding would not register within the acquired raw acceleration signal from the wheel-mounted accelerometer. Consequently, this would lead the AWOK algorithm to wrongfully flag, such portions of the trajectory as stationary segments of the signal. Whereas, in case of wheel slipping, the AWOK wheel-mounted accelerometer would register wheel cycles that did not result in vehicle motion. Hence, the faulty wheel cycles would result in erroneous distance measurement. Therefore, it is crucial to determine a way to remove such faulty wheel cycles from the distance measurements.

## Figures and Tables

**Table 1:** Results of comparing the AWOK-acquired distances against the reference total station distances.

Distance No.	Speed (km/h)	Reference Distance (m)	AWOK Distance (m)	Error (m)
1	40	193.8658	193.8933	−0.028
2	30	191.382	191.3073	0.075
3	20	190.6729	190.6592	0.014
4	10	190.6442	190.6821	−0.038
5	5	190.8821	191.0722	−0.190
			RMSE	0.042

**Table 2:** Results of comparing the AWOK-acquired distances against the reference of Global Navigation Satellite Systems (GNSS) results.

Total distance RMSE	11.029 m
% Error from RMSE	0.105%
% Error at the end of Trajectory	0.045%
AWOK on-track velocity RMSE	0.35 m/s
OBD2 on-track velocity RMSE	0.46 m/s

## Data Availability

The data presented in this study are available on request from the corresponding author.
